# Effects of probiotics on loperamide-induced constipation in rats

**DOI:** 10.1038/s41598-021-02931-7

**Published:** 2021-12-16

**Authors:** Takio Inatomi, Mihoko Honma

**Affiliations:** 1 Inatomi Animal Hospital, 1-1-24 Denenchofu, Ota-ku, Tokyo, 145-0071 Japan; 2 Kusama Animal Health Laboratory, 2240 Tsunehiro, Kashima-shi, Saga, 849-1301 Japan

**Keywords:** Applied microbiology, Therapeutics, Quality of life

## Abstract

The role of probiotics in mitigating constipation, gut immunity, and gut microbiota has not been well studied. We aimed to evaluate the effects of probiotics on loperamide (LP)-induced constipation in Sprague–Dawley rats. Altogether, 150 male Sprague–Dawley rats (age 8 weeks) were used in the experiments following a 12-day acclimatisation period and were randomly divided into three treatment groups (groups 1, 2, and 3). Spastic constipation was induced via oral LP administration (3 mg/kg) for 6 days, 1 h before administering each test compound in groups 1 and 2. A probiotic solution (4 mL/kg body weight) was orally administered once a day for 6 days in group 2. In group 1, a phosphate buffer solution was orally administered once a day for 6 days, 1 h after each LP administration. In group 3, a phosphate buffer solution was orally administered once a day for 6 days. In the probiotic group, faecal parameters improved; faecal n-butyric acid, acetic acid, and IgA concentrations were increased; intestinal transit time was shortened; and disturbance of intestinal microbiota was inhibited. Our findings suggest that this probiotic was useful in improving various symptoms caused by constipation.

## Introduction

Constipation is a common problem, and probiotics have been reported to improve bowel motility^1,2^. Symptoms of constipation include a decrease in the frequency of bowel movements and amount of faeces, painful bowel movements, dry faeces, and dissatisfaction after defecation^3^. Constipation has a significant impact on an individual’s quality of life, and prebiotics and probiotics are expected to help in the treatment of this condition^4,5^. Therefore, there is growing interest in better understanding the effects of probiotics on constipation.

Probiotics are defined as live microorganisms that, when administered in adequate amounts, confer a health benefit on the host^6^. Probiotics may, in fact, facilitate a return to the normal status after a perturbation of the microbiota (e.g., because of the use of antibiotics or illness) or may reduce the degree of change invoked by such situations. A few studies have measured a probiotic-enhanced return to baseline levels after antibiotic use in humans^7,8^. The concept of stabilising intestinal microorganisms via the associated probiotics for health improvement dates to the beginning of the last century. Several studies have been conducted to determine the effects of probiotic cultures on health^9–14^. The ingestion of probiotics is beneficial for treating different diarrhoea-like disorders and lowering the levels of metabolites that are harmful to health, including cancerous markers in the colon^15,16^. Probiotic microorganisms promote various immunomodulatory effects by modulating the gut microbiota^17,18^. Thus, probiotic bacteria could be used for the treatment of constipation because of their health-promoting benefits.

Recently, the increasing number of immunocompromised patients has posed a problem; some patients have contracted opportunistic infections caused by some bacterial species, which are considered non-pathogenic bacteria^19^. However, there are only few studies on intestinal immunity during constipation^20^. Therefore, determining the safety of probiotics is crucial.

Probiotics containing *Bacillus subtilis* TO-A, *Enterococcus faecium* T-110, and *Clostridium butyricum* TO-A (Bio-three, TOA Biopharma Co., Ltd, Tokyo, Japan) are widely utilised for the treatment and prevention of infectious diseases in Japan, China, and India. NOGUCHI et al. reported that *E. faecium* T-110 (In the paper, *E. faecium* T-110 is described as TP1240) has no virulence genes, is clearly genetically different from clinical isolates, and is unlikely to be a causative agent of opportunistic infections^21^. Takeshi et al. reported that *Clostridium butyricum* TO-A does not contain a toxin-producing gene^22^. Purushothaman et al. reported that *Bacillus subtilis* TO-A does not contain major genes for pathogenicity and antibiotic resistance^23^. With respect to safety, the probiotics containing *B. subtilis* TO-A, *E. faecium* T-110, and *C. butyricum* TO-A are considered suitable for treating constipation.

Several studies on the role of probiotics in the mitigation of constipation have been conducted. However, to the best of our knowledge, only few studies have investigated probiotics in the mitigation of constipation and gut immunity. Therefore, this study aimed to investigate the effects of probiotics on constipation relief, gut immunity, and gut microbiota in a rat model of constipation.

## Results

Changes in body weight are shown in Table 1. No differences in body weight were observed among the three groups (group1: placebo group, group2: probiotic group, group3: control group) in this study. The faecal parameters are presented in Table 2. At 1 day before the 24-h treatment (day 0), there were no differences in the pellet numbers, wet and dry weights of faeces, or water contents of faeces among the three groups. However, after the fourth and sixth administrations for 24 h (day 4 and day 6), these parameters were all significantly higher in groups 2 and 3 than in group 1 (both *p* < 0.01). The SCFA concentrations in faeces are shown in Table 3. At day 0, there were no differences in the SCFA concentrations in the faeces among the three groups; however, on days 4 and 6, the concentrations of n-butyric acid and acetic acid were significantly higher in groups 2 and 3 than in group 1 (both *p* < 0.01).

**Table 1:** Body weight (g).

	Day 0^a^	Day 4^a^	Day 6^a^
Group 1^b^ (placebo)	290.1 ± 1.641	293.4 ± 1.733	290.9 ± 2.236
Group 2^b^ (probiotics)	292.5 ± 1.552	290.3 ± 1.879	288.5 ± 1.941
Group 3^b^ (control)	289.7 ± 0.263	289.3 ± 1.578	289.5 ± 1.368

^a^Day 0: 1 day before the first dose of test compound; Day 4: 24 h after the fourth dose; Day 6: 24 h after the sixth dose.

^b^N = 50.

**Table 2:** Faecal parameters on day 0^a^, 4^b^ and 6^c^.

	Pellet number (numbers/rat)	Wet weight of faeces (g/rat)	Dry weight of faeces (g/rat)	Water content of faeces (%/rat)
Group1^f^ (day 0) (placebo)	58.0 ± 0.42	9.41 ± 0.15	6.49 ± 0.11	31.0 ± 0.29
Group2^f^ (day 0) (probiotics)	57.7 ± 0.45	9.34 ± 0.15	6.38 ± 0.09	31.6 ± 0.36
Group3^f^ (day 0) (control)	57.5 ± 0.34	9.27 ± 0.16	6.41 ± 0.11	30.8 ± 0.17
Group1^f^ (day 4) (placebo)	29.5 ± 0.20^d^	4.12 ± 0.05^d^	3.35 ± 0.04^d^	13.9 ± 0.39^d^
Group2^f^ (day 4) (probiotics)	57.8 ± 0.29^e^	9.01 ± 0.10^e^	6.24 ± 0.04^e^	30.3 ± 0.80^e^
Group3^f^ (day 4) (control)	57.8 ± 0.41^e^	9.00 ± 0.14^e^	6.30 ± 0.10^e^	30.0 ± 0.18^e^
Group1^f^ (day 6) (placebo)	29.4 ± 0.25^d^	4.19 ± 0.03^d^	3.63 ± 0.03^d^	13.3 ± 0.29^d^
Group2^f^ (day 6) (probiotics)	55.3 ± 0.32^e^	9.10 ± 0.07^e^	6.35 ± 0.07^e^	30.2 ± 0.62^e^
Group3^f^ (day 6) (control)	56.0 ± 0.29^e^	8.86 ± 0.10^e^	6.18 ± 0.09^e^	30.2 ± 0.72^e^

^a^Day 0: 1 day before the first dose of test compound.

^b^Day 4: 24 h after the fourth dose.

^c^Day 6: 24 h after the sixth dose.

^d, e^Different letters within columns indicate differences among treatment groups at same day. (p < 0.05).

^f^N = 50.

**Table 3:** Short-chain fatty acid concentration of faeces (µmol/g) on day 0^a^, 4^b^ and 6^c^.

	n-butyric acid	Acetic acid	Propionic acid	Minor acids	Total SCFA
Group1^f^ (day 0) (placebo)	5.635 ± 0.086	24.66 ± 0.265	9.549 ± 0.182	6.432 ± 0.637	46.28 ± 0.596
Group2^f^ (day 0) (probiotics)	5.546 ± 0.075	25.30 ± 0.243	9.318 ± 0.249	5.158 ± 0.552	45.32 ± 0.620
Group3^f^ (day 0) (control)	5.585 ± 0.018	25.00 ± 0.019	9.557 ± 0.255	6.482 ± 0.706	46.62 ± 0.795
Group1^f^ (day 4) (placebo)	5.088 ± 0.076^d^	24.65 ± 0.253^d^	8.815 ± 0.148	3.894 ± 0.384	42.45 ± 0.285
Group2^f^ (day 4) (probiotics)	5.936 ± 0.082^e^	25.99 ± 0.329^e^	9.036 ± 0.418	1.655 ± 0.208	42.62 ± 0.298
Group3^f^ (day 4) (control)	5.912 ± 0.015^e^	25.89 ± 0.047^e^	8.633 ± 0.157	2.693 ± 0.240	43.13 ± 0.294
Group1^f^ (day 6) (placebo)	5.101 ± 0.081^d^	24.90 ± 0.185^d^	9.876 ± 0.217	3.574 ± 0.343	43.45 ± 0.270
Group2^f^ (day 6) (probiotics)	6.033 ± 0.079^e^	25.82 ± 0.265^e^	9.278 ± 0.313	1.853 ± 0.295	42.99 ± 0.290
Group3^f^ (day 6) (control)	6.207 ± 0.095^e^	26.00 ± 0.275^e^	9.699 ± 0.323	1.020 ± 0.141	42.92 ± 0.258

^a^Day 0: 1 day before the first dose of test compound.

^b^Day 4: 24 h after the fourth dose.

^c^Day 6: 24 h after the sixth dose.

^d, e^Different letters within columns indicate differences among treatment groups at same day (p < 0.05).

^f^N = 50.

There were no significant differences between the groups at any collection period in terms of total SCFA, propionic acid, or minor acids (i-butyric acid + i-valeric acid + n-valeric acid). The faecal immunoglobulin (Ig) A concentrations are shown in Table 4. At day 0, there were no differences in the faecal IgA concentrations among the three groups, whereas on days 4 and 6, the concentrations were significantly higher in groups 2 and 3 than in group 1 (both *p* < 0.01). The gastrointestinal transit times are shown in Table 5. The gastrointestinal transit times were significantly shorter in groups 2 and 3 than in group 1 (*p* < 0.01). The amount of *Bifidobacterium* sp*., Lactobacillus/Enterococcus* spp., *Bacteroides* spp., *Clostridium* spp., *Clostridium coccoides-Eubacterium rectale* group, and *E. coli* spp., in the faeces are shown in Table 6. On day 0, the amount of *Bifidobacterium* sp*.*, *Lactobacillus/Enterococcus* spp., *Bacteroides* spp., *Clostridium* spp., *Clostridium coccoides-Eubacterium rectale* group, and *E. coli* spp., were not significantly different among the three groups. On days 4 and 6, the amount of *Bacteroides* spp., and *E. coli* spp., were higher in group 1 than in groups 2 and 3, whereas the amount of *Bifidobacterium* sp*., Lactobacillus/Enterococcus* spp.*,* and *Clostridium coccoides-Eubacterium rectale group* were lower in group 1 than in groups 2 and 3 (*p* < 0.01).

**Table 4:** Faecal IgA concentration (mg/g).

	Day 0^a^	Day 4^a^	Day 6^a^
Group 1^d^ (placebo)	2.046 ± 0.038	2.037 ± 0.041^b^	1.971 ± 0.036^b^
Group 2^d^ (probiotics)	2.044 ± 0.044	2.363 ± 0.028^c^	2.285 ± 0.035^c^
Group 3^d^ (control)	2.098 ± 0.028	2.276 ± 0.048^c^	2.271 ± 0.025^c^

^a^Day 0: 1 day before the first dose of test compound; Day 4: 24 h after the fourth dose; Day 6: 24 h after the sixth dose.

^b, c^Different letters within columns indicate differences among treatment groups (*p* < 0.05).

^d^N = 50.

**Table 5:** Gastrointestinal transit time (min) in rats with constipation induced by LP.

	Gastrointestinal transit time
Group 1^c^ (placebo)	659.3 ± 2.1^a^
Group 2^c^ (probiotics)	399.4 ± 2.0^b^
Group 3^c^ (control)	395.3 ± 1.8^b^

^a,b^Different letters within columns indicate differences among treatment groups (*p* < 0.05).

^c^N = 50.

**Table 6:** Microbiological analyses of faeces on day 0^a^, 4^b^ and 6^c^ (log cells/g).

	Bif164	Lab158	Bac303	Chis150	Erec482	EC 1531	DAPI
Group1^f^ (placebo) (day 0)	10.17 ± 0.02	10.11 ± 0.04	9.84 ± 0.04	9.99 ± 0.08	10.22 ± 0.04	9.77 ± 0.03	11.73 ± 0.02
Group2^f^ (probiotics) (day 0)	10.21 ± 0.02	10.03 ± 0.05	9.79 ± 0.05	10.06 ± 0.06	10.21 ± 0.04	9.77 ± 0.03	11.73 ± 0.02
Group3^f^ (control) (day 0)	10.19 ± 0.02	10.08 ± 0.05	9.75 ± 0.05	10.02 ± 0.07	10.19 ± 0.04	9.80 ± 0.02	11.73 ± 0.02
Group1^f^ (placebo) (day 4)	9.65 ± 0.04^d^	9.77 ± 0.05^d^	10.07 ± 0.07^d^	9.77 ± 0.06	9.77 ± 0.03^d^	10.22 ± 0.04^d^	11.14 ± 0.01
Group2^f^ (probiotics) (day 4)	10.14 ± 0.05^e^	10.12 ± 0.05^e^	9.65 ± 0.04^e^	9.80 ± 0.06	10.23 ± 0.04^e^	9.69 ± 0.04^e^	11.14 ± 0.01
Group3^f^ (control) (day 4)	10.23 ± 0.06^e^	10.05 ± 0.05^e^	9.67 ± 0.04^e^	9.88 ± 0.06	10.17 ± 0.05^e^	9.69 ± 0.03^e^	11.16 ± 0.01
Group1^f^ (placebo) (day 6)	9.66 ± 0.04^d^	9.65 ± 0.05^d^	9.94 ± 0.06^d^	9.63 ± 0.05	9.64 ± 0.05^d^	10.06 ± 0.04^d^	11.27 ± 0.05
Group2^f^ (probiotics) (day 6)	10.10 ± 0.03^e^	10.09 ± 0.04^e^	9.67 ± 0.05^e^	9.56 ± 0.06	10.26 ± 0.04^e^	9.61 ± 0.04^e^	11.29 ± 0.04
Group3^f^ (control) (day 6)	10.12 ± 0.03^e^	10.06 ± 0.04^e^	9.69 ± 0.05^e^	9.64 ± 0.06	10.19 ± 0.03^e^	9.57 ± 0.04^e^	11.32 ± 0.04

^a^Day 0: 1 day before the first dose of test compound.

^b^Day 4: 24 h after the fourth dose.

^c^Day 6: 24 h after the sixth dose.

^d, e^Different letters within columns indicate differences among treatment groups at same day (p < 0.05). ^f^N= 50.

## Discussion

Loperamide (LP)-induced delay in colonic transit occurs due to decreased stool frequency and increased colonic contractions in humans^24^. This drug inhibits intestinal water secretion and colonic peristalsis^25^, which extends the faecal evacuation time and delays intestinal luminal transit^26^. Thus, LP-induced constipation is considered a model of spastic constipation^27^.

In the present study, oral administration of probiotics containing *B. subtilis* TO-A, *E. faecium* T-110, and *C. butyricum* TO-A improved faecal parameters, faecal SCFA concentration, faecal IgA concentration, gastrointestinal transit time, and gut microbiota. Constipation can arise due to various causes, including dietary habits, use of chemical compounds (e.g., morphine), and psychological stress^28^. In this study, the dose of the probiotics containing *B. subtilis* TO-A, *E. faecium* T-110 and *C. butyricum* TO-A, which were used in rats, was determined based on the dose used in humans^29^. The colony forming unit (CFU)/rat weight (g) of the administered probiotics were as follows: *B. subtilis* TO-A 2.5 × 10^3^, *E. faecium* T-110 1 × 10^4^, and *C. butyricum* TO-A 2.5 × 10^3^/g. Successful colonization of probiotic bacteria in the gut environment is an important factor for their function^30^. In this study, we could not confirm colonization of this probiotic in the intestine because the identification method was not disclosed by the manufacturer, but previous studies have confirmed the colonization of probiotics in the intestine^30,31^.

The administration of probiotics containing *B. subtilis* TO-A, *E. faecium* T-110 and *C. butyricum* TO-A did not lead to any adverse effects in rats. In group 1, the results of the analysis of faecal parameters obtained 24 h after the fourth administration suggested that constipation was properly induced using LP, in accordance with the findings of previous studies^32–35^. The bodyweight of rats was not markedly different between groups 1 and 2 in this study, consistent with the findings of previous studies^32,33^.

Shi et al. reported significantly lower faecal levels of SCFA (acetic acid, propionic acid, and butyric acid) in a population with constipation than that in a healthy population^36^. These observations indicate an association between the occurrence of constipation and the intestinal levels of SCFAs. Therefore, SCFAs produced by intestinal microbiota or probiotics were believed to be effective for constipation alleviation. Moreover, administration of *Lactobacillus plantarum* NCU116 had been reported to significantly improve the constipation symptoms in mice and lead to significant increases in acetic and propionic acid levels in their faeces^37^.

The mechanisms by which SCFA affect constipation include stimulation of the intestinal tract and enhancement of its motility^38–40^. In the present study, probiotics containing *B. subtilis* TO-A, *E. faecium* T-110, and *C. butyricum* TO-A significantly increased faecal concentrations of butyric and acetic acids in a rat model of LP-induced constipation. These results are consistent with those of our previous study^41^. Considering the results of previous studies, the findings of the present study suggest that the three bacteria contained in the probiotics produced SCFA. In this study, probiotics containing *B. subtilis* TO-A, *E. faecium* T-110, and *C. butyricum* TO-A significantly improved the faecal parameters, including pellet numbers, wet weights of faeces, dry weights of faeces, and water contents of faeces. From the abovementioned results, it is considered that the administration of probiotics containing *B. subtilis* TO-A, *E. faecium* T-110, and *C. butyricum* TO-A improved constipation symptoms by increasing SCFA production, which consequently improved faecal parameters. Improvement in constipation symptoms may have improved the gastrointestinal transit time.

Havenaar and Spanhaak showed that probiotics stimulate immunity in animals in the following two ways: (1) microbiota from probiotics migrate throughout the gut wall and multiply to a limited extent; and (2) antigens released by dead microorganisms are absorbed, thereby stimulating the immune system^42^. IgA plays an important role in intestinal immunity by binding to and neutralising pathogens and toxins in the intestinal tract^43^. Probiotics containing *B. subtilis* TO-A, *E. faecium* T-110, and *C. butyricum* TO-A cause an increase in IgA production in the mesenteric lymph nodes in rats^44^ and an increase in IgA concentrations in the jejunum and ileum in broiler chickens^45^. Heat-killed *E. faecium* T-110 supplementation increases IgA concentrations in the faeces of hamsters^46^. In the present study, administration of probiotics increased the concentration of faecal IgA. In line with previous studies, our findings suggest that antigens released from the cell components of the administered probiotic microorganisms were absorbed, stimulated the immune system, increased IgA production, and increased faecal IgA concentrations. However, the detailed mechanisms of action are unknown and further investigation into the immunostimulatory effect of cell components is needed.

Constipation is known to affect the intestinal microbiota by decreasing beneficial bacteria and increasing harmful bacteria^37^. In the present study, LP-induced constipation in rats caused a decrease in the amount of *Bifidobacterium* sp*.*, *Lactobacillus/Enterococcus* spp., and *Clostridium coccoides-Eubacterium rectale group* and an increase in the number of *Bacteroides* spp., and *E. coli* spp. in the faeces. This result is consistent with that of previous reports^47–49^. On the other hand, in LP-induced rats treated with probiotics containing *B. subtilis* TO-A, *E. faecium* T-110, and *C. butyricum* TO-A, there was no change in the amounts of *Bifidobacterium* sp., *Lactobacillus/Enterococcus* spp., *Clostridium coccoides-Eubacterium rectale group, Bacteroides* spp., or *E. coli* spp. in the faeces. *B. subtilis* TO-A promotes the growth of *Bifidobacterium*^50^*.* Bifidobacteria have a more efficient glycolytic system and efficiently produce lactic acid and acetic acid^51^. Moreover, lactic acid and acetic acid produced by bifidobacteria are substrates for butyrate-producing colonic bacteria and stimulate their growth^52^. This study suggests that the bifidobacteria increased by probiotic administration efficiently produced acetic acid, thereby increasing levels of the *Clostridium coccoides-Eubacterium rectale group*, a group of butyrate-producing bacteria that use acetic acid as a substrate. In general, *Bacillus subtilis* is known to produce amylase, which promotes the saccharification of starch that is not available to *lactobacilli/enterococci*, thereby increasing the substrate available to *lactobacilli/enterococci*^53,54^. The present study suggests that *Bacillus subtilis* TO-A in the probiotic increases the number of *Lactobacillus/Enterococcus* by increasing levels of the substrate. *E. faecium* T-110 and *C. butyricum* TO-A inhibit the growth of *E. coli*^55^. It is not clear as to why administration of this probiotic results in the reduction of *Bacteroides* spp. in this study. Further research is needed in this regard. Consistent with the findings of previous studies, our findings suggest that the administration of probiotics containing *B. subtilis* TO-A, *E. faecium* T-110, and *C. butyricum* TO-A prevented the dysbiosis in intestinal microbiota caused by constipation. In this study, we investigated the intestinal microbiota using the FISH method, however, it is believed that analysis of the intestinal microbiota using Next Generation Sequencing, which has a higher accuracy, will be necessary in the future.

This study has some limitations. First, this study did not examine the effect of probiotics on recurrent constipation. In humans, constipation is a recurring condition, and future longer-term studies are needed. Second, this study only investigated the effect of probiotics on spastic constipation. In the future, the effect of probiotics on constipation caused by other factors needs to be investigated. Finally, in this study, only faecal IgA was investigated as an index of intestinal immunity. More detailed investigations of the effect on immunity are needed in the future.

In conclusion, our results indicate that probiotics containing *B. subtilis* TO-A, *E. faecium* T-110, and *C. butyricum* TO-A increase the levels of intestinal SCFA especially butyric acid, thereby improving constipation. We also found that probiotics containing *B. subtilis* TO-A, *E. faecium* T-110, and *C. butyricum* TO-A stimulated the immune system, increased intestinal IgA, and improved gut microbiota. The results of this study show the potential of medical probiotics in improving constipation, immune status, and intestinal microbiota in immunocompromised infants, elderly, and patients, especially where safety is required. Further studies in humans are, therefore, needed.

## Materials and methods

### Ethical approval

All experimental procedures and animal care procedures were approved by the Ethics Committee of Kusama Animal Health Laboratory (Kashima, Japan; approval number 2020-003), performed in accordance with the fundamental guidelines for the proper conduct of animal experiments and related activities at academic research institutions under the jurisdiction of the Ministry of Education, Culture, Sports, Science, and Technology by the National Institutes of Health Guide for the Care and Use of Laboratory Animals, and reported as per the ARRIVE guidelines. The rats used in this study were kept in accordance with the guidelines for the care of laboratory animals until they died at the end of their lifespan without being used for any further studies.

### Experimental animals

A total of 150 male Sprague–Dawley rats (age, 8 weeks; Japan SLC, Inc., Hamamatsu, Japan) were used in the experiments following a 12-day acclimatisation period. They were randomly divided into three treatment groups (groups 1, 2, and 3), with 50 rats per group. The rats were individually housed in a polycarbonate cage inside a temperature (20–23 °C)- and humidity (40–50%)-controlled room. The light/dark cycle was 12/12 h, and basic diet (Rodent Diet CL-2; CLEA Japan, Tokyo, Japan) and water were supplied ad libitum. The probiotics (Bio-three, TOA Biopharma Co., Ltd, Tokyo, Japan, lot number: 5527) used in this study contained *B. subtilis* TO-A (5.0 × 10^7^ CFU g^−1^), *E. faecium* T-110 (2.0 × 10^8^ CFU g^−1^), and *C. butyricum* TO-A (5.0 × 10^7^ CFU g^−1^). The probiotics were dissolved in phosphate buffer solution (PBS) (FUJIFILM Wako Pure Chemical Corporation., Osaka, Japan) to a final concentration of 12.5 mg/mL. In group 1, PBS was orally administered once a day for 6 days at 1 h after each LP administration. In group 2, the probiotic solution (4 mL/kg body weight) was orally administered once a day for 6 days at 1 h after each LP (FUJIFILM Wako Pure Chemical Corporation., Osaka, Japan) administration, as suggested by previously reported studies^32,33^. In group 3, PBS was orally administered once a day for 6 days.

### Constipation induction in the rats

Constipation was induced in groups 1 and 2 via oral administration of 3-mg/kg LP once a day for 6 continuous days at 1 h before administration of each test material^32–35^.

### Changes in body weight

The body weights of the individual rats were measured daily, starting from the day before the administration of test compounds to the sixth day of the administration of test compounds and LP.

### Measurement of faecal parameters

Faeces excreted by the individual rats were collected 1 day before the first dose of the test compound (day 0), 24 h after the fourth dose (day 4), and 24 h after the sixth dose (day 6). The total number, water content, and wet weight of the faecal pellets were measured. The collected faecal pellets were dried at 60 °C in a general dry oven for 24 h to obtain the faecal dry weights.

### Short-chain fatty acid concentration in faeces

The short-chain fatty acid (SCFA) concentration in the faeces of each rat on days 0, 4, and 6 was measured using gas chromatography, as described previously^56^. Approximately 0.5 g of faeces from the dissections described above was gently squeezed into a micro-centrifuge tube (Funakoshi Co., Ltd., Tokyo, Japan) containing 1-mL 10% meta-phosphoric acid (FUJIFILM Wako Pure Chemical Corporation., Osaka, Japan) with 0.4-mL 4-methyl valeric acid (FUJIFILM Wako Pure Chemical Corporation., Osaka, Japan) per millilitre added as an internal standard. The solution was thoroughly vortexed and centrifuged at 5700×*g* for 20 min at 4 °C. The SCFA content of the supernatant was measured using an HP Agilent 6890 series gas chromatograph (Agilent Technologies Inc., Santa Clara, CA, USA) fitted with an HP 5973 series mass spectrometer (Agilent Technologies Inc., Santa Clara, CA, USA). The columns (Agilent Technologies Inc., Santa Clara, CA, USA) used were HP-free fatty acid polyester stationary phase capillary columns of polyethylene glycol on Shimalite TPA 60/80 (Shimadzu GLC Ltd., Tokyo, Japan) measuring 30-m long with a 0.25-mm internal diameter.

The parameters of gas chromatography were as follows: 1-µL injection volume, 240 °C injector temperature, 12.15-psi pressure, and 1.1-mL min^−1^ constant flow using helium (GL Sciences Inc., Tokyo, Japan) as a carrier. The fatty acids were eluted with the following oven programme: 80 °C initial temperature hold for 5 min, ramp 10 °C min^−1^ to 240 °C and hold for 12 min. The individual SCFA concentrations are expressed in µmol/g of wet faeces. In this study, short-chain fatty acids ranged from acetic acid, which has two carbons, to valeric acid, which has five carbons, and the total of these (six species, including isomers) was defined as total short-chain fatty acids.

### Faecal IgA concentration

The faecal IgA concentration in each rat was measured on days 0, 4, and 6 as described previously^57^. Faeces were suspended in six times the weight of PBS and extracted at 25 °C at 24 h. The extract was centrifuged at 1500×*g* for 10 min and the supernatant collected and stored at − 30 °C. Subsequently, the IgA concentration was measured using an enzyme-linked immunosorbent assay (ELISA) quantitation kit (Betyl Laboratories, Montgomery, Texas, USA). ELISA was conducted according to the manufacturer's protocol.

First, 100 µL of the sample or standard was added to the wells of the plate and allowed to stand at 22 °C for 1 h; the plate was then washed 4 times. Next, a rat IgA detection antibody was added to each well and allowed to stand at 22 °C for 1 h; it was then rinsed 4 times. Horseradish peroxidase solution A was allowed to stand at 22 °C for 30 min, and the plate was then rinsed 4 times. One hundred microliters of 3,3ʹ,5,5ʹ-tetramethylbenzidine substrate solution was added to each well, and the plate was developed in the dark for 30 min at 22 °C. After stopping the reaction by adding 100 µL of the stop solution to each well, the absorbance was measured at a 45-nm wavelength using a plate reader. Subsequently, a calibration curve was prepared, and the IgA concentration was calculated.

### Measurement of gastrointestinal transit time

The gastrointestinal transit time of the feed consumed by the rats was measured using a method described previously^58^. The animals were housed in the fasted state for 12 h after day 6. Rats were then fed a diet mixed with 1 g of 10% Coomassie brilliant blue dye (FUJIFILM Wako Pure Chemical Corporation., Osaka, Japan), and the total time taken to defecate the blue-coloured faecal pellets was determined to be the gastrointestinal transit time.

### Enumeration of bacterial populations in faeces by FISH (fluorescence in situ hybridization)

FISH was performed essentially as described by Martín-Peláez et al.^59^. Faeces were immediately moved into a sterile microtube and mixed with 0.1 M PBS, pH 7.4, at a concentration of 10% (W/V). The slurry was blended and filtered in the stomacher bag for 2 min. 500 µL of faecal samples were fixed in three volumes of ice-cold 4% (w/v) paraformaldehyde (FUJIFILM Wako Pure Chemical Corporation., Osaka, Japan) for 4 h at 4 °C. They were then centrifuged at 13,000×*g* for 5 min and washed twice in 1 mL of sterile PBS. The cells were pelleted by centrifugation and resuspended in 150 µL of sterile PBS, to which 150 µL of ethanol (FUJIFILM Wako Pure Chemical Corporation., Osaka, Japan) was added. The samples were then vortexed and stored at − 20 °C until used in hybridizations. For the hybridizations, 20 µL of each sample was pipetted onto Teflon- and poly-l-lysine-coated, six-well (10 mm diameter each) slides (Tekdon Inc., Myakka City, USA). The samples were dried onto the slides at 46 °C for 15 min and afterwards dehydrated in an alcohol series (50%, 80%, and 96%, for 3 min each). The ethanol was allowed to evaporate from the slides before the probes were applied to the samples. To permeabilize the cells for use with probes Lab158 and Rfla729/Rbro730, samples were treated with 50 µL of lysozyme (1 mg mL^−1^ in 100 mM Tris–HCl, pH 8.0) (FUJIFILM Wako Pure Chemical Corporation, Osaka, Japan) at 37 °C for 15 min before being washed briefly (2–3 s) in water and afterwards dehydrated in the ethanol series. A probe/hybridization buffer mixture (5 µL of a 50 ng µL^−1^ stock of probe plus 45 µL of hybridization buffer) was applied to the surface of each well. Hybridizations were performed for 4 h in an ISO20 oven (Boekel Scientific, Pennsylvania, USA). For the washing step, slides were placed in 50 mL of wash buffer containing 20 µL of 4ʹ,6-diamidino-2-phenylindole dihydrochloride (DAPI; 50 ng µL^−1^; Sigma, Tokyo, Japan) for 15 min. They were then briefly washed (2–3 s) in ice-cold water and dried under a stream of compressed air. 5 µL of antifade reagent (polyvinyl alcohol mounting medium with DABCO™ antifading; Sigma, Tokyo, Japan) was added to each well and a coverslip was applied. Slides were stored in the dark at 4 °C (for a maximum of 3 days) until cells were counted under a Nikon E400 Eclipse microscope. DAPI-stained slides were visualized with the aid of a DM 400 filter and probe slides with the aid of a DM 575 filter. Numbers of specific bacteria and DAPI-stained entities (used to count total bacteria) were determined using the following equation:

$${\text{DF }} \times {\text{ ACC }} \times { 6732}.{42 } \times { 5}0 \, \times {\text{ DF}}_{{{\text{sample}}}} ,$$

where DF is the dilution factor (300/500 = 0.6), ACC is the average cell count of 15 fields of view and DF_sample_ refers to the dilution of sample used with a particular probe or stain (e.g. 50× for Bif164 counts). The figure 6732.42 refers to the area of the well divided by the area of the field of view and the factor 50 takes the cell count back to per millilitre of sample. The units were converted from per mL to per gram, and the amount of each bacterium was expressed per gram of wet faeces. All probes were Cy3-labelled and synthesized by Sigma Aldrich Japan (Tokyo, Japan). Table 7 gives the details of the probes used in this study^60–64^.

**Table 7:** Probes used for FISH analysis of bacterial populations in faeces.

Short name	Accession no.	Full name	Target species	Temperature (°C)		Sequence (5ʹ to 3ʹ)	References
				Hybridization	Washing		
Bif164	pB-00037	S-G-Bif-0164-a-A-18	Most *Bifidobacterium* spp. and Parascardovia denticolens	50	50	CATCCGGCATTACCACCC	Langendijk et al.^60^
Lab158	ND	S-G-Lab-0158-a-A-20	Most Lactobacillus, Leuconostoc and *Weissella* spp.; *Lactococcus lactis*; all Vagococcus, Enterococcus, Melisococcus, Tetragenococcus, Catellicoccus, Pediococcus and *Paralactobacillus* spp.	50	50	GGTATTAGCAYCTGTTTCCA	Harmsen et al.^61^
Bac303	pB-00031	S-*-Bacto0303-a-A-17	Most Bacteroides sensu stricto and *Prevotella* spp.; all Parabacteroides; Barnesiella viscericola and Odoribacter splanchnicus	46	48	CCAATGTGGGGGACCTT	Manz et al.^62^
Chis150	pB-00962	S-*-Chis0150-a-A-23	Most members of Clostridium cluster I; all members of Clostridium cluster II; *Clostridium tyrobutyricum*; *Adhaeribacter aquaticus* and Flexibacter canadensis (family Flexibacteriaceae); [Eubacterium] combesii (family Propionibacteriaceae)	50	50	TTATGCGGTATTAATCTYCCTTT	Franks et al.^63^
Erec482	pB-00963	S-*-Erec0482-a-A-19	Most members of Clostridium cluster XIVa; Syntrophococcus sucromutans, [Bacteroides] galacturonicus and [Bacteroides] xylanolyticus, Lachnospira pectinschiza and *Clostridium saccharolyticum*	50	50	GCTTCTTAGTCARGTACCG	Franks et al.^63^
EC 1531	pB-3938	L-S-Eco-1531-a-A-21	E. coli spp.	37	37	CACCGTAGTGCCTCGTCATCA (23S rRNA)	Poulsen et al.^64^

Probe designation according to Alm et al. ^65^ This information was retrieved from probeBase.

These probes were used together in equimolar concentrations (both at 50 ng µL^−1^).

Formamide (20%) was included in the hybridization buffer.

*ND, No information relating to these probes has been deposited in probeBase (http://www.microbial-ecology.net/probebase).

### Statistical analyses

We determined the sample size from the method described by Cohen^66^ (Effect size = 0.25, Power = 0.8, α = 0.05), and N was determined using G*Power^67^. The different dose groups were compared using multiple comparison tests. The Bartlett test was performed to examine variance homogeneity. When no significant deviation was observed with the Bartlett test, one-way analysis of variance was conducted to evaluate differences in the means among the three groups, and the significance of the intergroup mean differences was tested using Bonferroni’s test. If a significant deviation from variance homogeneity was observed, a non-parametric comparison test, the Kruskal–Wallis H test, was performed. In cases of significant differences in the Kruskal–Wallis H test, the Mann–Whitney U test was performed to examine the significantly different pairs. Results were considered significant at *p* < 0.05. The statistical analyses were performed using EZR software (Saitama Medical Center, Jichi Medical University); EZR is a graphical user interface for R (The R Foundation for Statistical Computing, version 2.13.0).

## Data Availability

The data that support the findings of this study are available from the corresponding author (T.I.) upon reasonable request.
